# LncRNA DNM3OS regulates GREM2 via miR-127-5p to suppress early chondrogenic differentiation of rat mesenchymal stem cells under hypoxic conditions

**DOI:** 10.1186/s11658-021-00269-6

**Published:** 2021-05-28

**Authors:** Xiaozhong Zhou, Wangyang Xu, Yeyang Wang, Hui Zhang, Li Zhang, Chao Li, Shun Yao, Zixiang Huang, Lishan Huang, Dixin Luo

**Affiliations:** 1 The Spine Department, Orthopaedic Center, Guangdong Second Provincial General Hospital, No. 466, Xingangzhong Road, Haizhu District, Guangzhou, 510317 Guangdong People’s Republic of China; 2 The Second School of Clinical Medicine, Southern Medical University, Guangzhou, China

**Keywords:** Mesenchymal stem cells, Chondrogenic differentiation, Hypoxic condition, miR-127-5p, GREM2, DNM3OS

## Abstract

**Background:**

Improved chondrogenic differentiation of mesenchymal stem cells (MSCs) by genetic regulation is a potential method for regenerating articular cartilage. MiR-127-5p has been reported to promote cartilage differentiation of rat bone marrow MSCs (rMSCs); however, the regulatory mechanisms underlying hypoxia-stimulated chondrogenic differentiation remain unknown.

**Methods:**

rMSCs were induced to undergo chondrogenic differentiation under normoxic or hypoxic conditions. Expression of lncRNA DNM3OS, miR-127-5p, and GREM2 was detected by quantitative real-time PCR. Proteoglycans were detected by Alcian blue staining. Western blot assays were performed to examine the relative levels of GREM2 and chondrogenic differentiation related proteins. Luciferase reporter assays were performed to assess the association among DNM3OS, miR-127-5p, and GREM2.

**Results:**

MiR-127-5p levels were upregulated, while DNM3OS and GREM2 levels were downregulated in rMSCs induced to undergo chondrogenic differentiation, and those changes were attenuated by hypoxic conditions (1% O_2_). Further in vitro experiments revealed that downregulation of miR-127-5p reduced the production of proteoglycans and expression of chondrogenic differentiation markers (COL1A1, COL2A1, SOX9, and ACAN) and osteo/chondrogenic markers (BMP-2, p-SMAD1/2). MiR-127-5p overexpression produced the opposite results in rMSCs induced to undergo chondrogenic differentiation under hypoxic conditions. GREM2 was found to be a direct target of miR-127-5p, which was suppressed in rMSCs undergoing chondrogenic differentiation. Moreover, DNM3OS could directly bind to miR-127-5p and inhibit chondrogenic differentiation of rMSCs via regulating GREM2.

**Conclusions:**

Our study revealed a novel molecular pathway (DNM3OS/miR-127-5p/GREM2) that may be involved in hypoxic chondrogenic differentiation.

## Background

Osteoarthritis (OA) is the most prevalent chronic joint disease in older individuals and a leading cause of disability worldwide [1]. OA is characterized by degeneration of articular cartilage, usually following trauma and excessive mechanical stress [2, 3]. Although progress has been made in conventional interventions for OA, including the injection of pain-relieving drugs and surgical arthroplasty, these therapies are relatively costly and inefficient, making them unsatisfactory options for treating OA [4–6]. Notably, the regeneration of articular cartilage and bone has recently been suggested as a promising therapeutic approach [7]. However, the lack of self-restoration ability makes it difficult to repair a cartilage defect [8].

Mesenchymal stem cells (MSCs) are a kind of cells with the ability of multi-differentiation, and could possibly be used for cell-based regenerative therapy of articular cartilage defects [9]. In addition to growth factors, cytokines, and 3-dimensional scaffolds [10, 11], oxygen levels and oxygen tension are other important regulators of MSC differentiation [12, 13]. Some studies have demonstrated that hypoxic conditions promote chondrogenic differentiation and increase cartilage protein synthesis by upregulating SOX9, type II collagen, and aggrecan [14, 15]. In contrast, persistent exposure of MSCs to hypoxia can downregulate their levels of CBFA-1/Runx2, osteocalcin, and type I collagen [16]. These findings regarding the role played by hypoxia in MSC differentiation remain controversial because the relevant studies used different hypoxia exposure times and sources of MSCs. However, those studies prompted us to explore the mechanisms that regulate the differentiation of MSCs into chondrocytes under hypoxic conditions.

MicroRNAs (miRNAs/miRs) comprise a class of small endogenous and conserved non-coding RNA molecules with a length of 21–25 nucleotides. MiRNAs regulate the function of downstream mRNAs by recognizing and binding to a specific site in the 3′-untranslated regions (3ʹUTR) of regulated genes [17]. Accumulating evidence suggests that certain aberrantly expressed miRNAs (e.g., miR-410 [18], miR-134 [19], and miR-146b [20]) participate in regulating the chondrogenic differentiation of MSCs. In recent years, miR-127-5p has been reported to be associated with the development of OA. Zhou et al. [21] reported that circRNA.33186 maintains MMP-13 expression by sponging miR-127-5p, which plays an important role in OA pathogenesis. Park et al. [22] found that expression of MMP-13 is inhibited by miR-127-5p in human chondrocytes. The role played by miR-127-5p in OA progression has been investigated by Tu et al. [23] and Liang et al. [24]. It was also reported that miR-127-5p alleviates cartilage hypertrophy by promoting chondrogenic differentiation [25].

Gremlin2 (GREM2) is a secreted bone morphogenic protein (BMP) antagonist, which is needed for chondrogenic differentiation [26]. Wang et al. [27] demonstrated that knockdown of GREM2 increased the BMP-2-promoted osteogenic differentiation of human MSCs (hMSCs) by activating the downstream pathway. It has been reported that long noncoding RNAs (lncRNAs) contain miRNA-response elements (MREs) that function as competing endogenous RNAs (ceRNAs), and play key roles in various pathological processes, including OA development [28–30]. Our previous bioinformatic analysis not only predicted GREM2 as the target of miR-127-5p, but also identified lncRNA dynamin 3 opposite strand (DNM3OS) as an endogenous sponge of miR-127-5p that could directly bind to miR-127-5p in a sequence-specific manner. Nevertheless, the function and regulatory mechanism of DNM3OS/miR-127-5p/GREM2 in chondrogenic differentiation under hypoxic conditions have not been elucidated.

In the present study, we first constructed a cell model of chondrogenic differentiation of bone marrow rat mesenchymal stem cells (rMSCs) under hypoxic conditions, and then analyzed the model cells for DNM3OS, miR-127-5p, and GREM2 expression. Next, we investigated the regulatory role played by DNM3OS/miR-127-5p/GREM2 in the chondrogenic differentiation process and validated the association between miR-127-5p and GREM2 or DNM3OS. Our results provide novel insights into the mechanism by which MSCs differentiate under hypoxic conditions.

## Materials and methods

### Isolation and culture of rMSCs

Bone marrow rMSCs were isolated from six-week-old male Norwegian brown rats (weight range, 100–120 g) purchased from the University of South China. In brief, the rats were sacrificed by controlled inhalation of CO_2_ and their bone marrow was flushed from the hind legs with low-glucose DMEM containing 10% fetal bovine serum (FBS). Next, the rMSCs were collected and incubated in a 10-cm diameter culture plate until they reached 80–90% confluence. After being detached from the plate by treatment with 0.25% trypsin, purified fourth-generation rMSCs were acquired and added to 10% FBS DMEM medium for subsequent analysis. All animal experiments have met with approval of Animal Care and Use Guidelines of the Second General Hospital of Guangdong Province (AE-20191215).

### Hypoxia intervention

For hypoxic culture, rMSCs were cultured in a tri-gas incubator containing 1% O_2_, 94% N_2_, and 5% CO_2_ at 37 °C for time periods of 24, 48 h, 7 days and 14 days. As controls, rMSCs were cultured under normoxic conditions (21% O_2_, 74% N_2_, 5% CO_2_, 37 °C) for 24, 48 h, 7 days, and 14 days.

### Chondrogenic differentiation

To induce chondrogenic differentiation, rMSCs were cultured in MSC chondrogenic differentiation medium (MCDM) under normoxic or hypoxic conditions for 7 and 14 days. The inductive medium was renewed every third day.

### Cell activity assay

The rMSCs were cultured with MCDM under hypoxic conditions (1, 2, 4, 8, 16, 21% O_2_) for 14 days and the cell activity was assessed using Cell Counting Kit-8 (Dojindo, Japan).

### Cell transfection and groups

A small interfering RNA targeting SHP (si-SHP) and DNM3OS (si-DNM3OS), miR-127-5p mimics, an inhibitor, and a negative control (NC) were each synthesized by RiboBio (Guangzhou, China). A DNM3OS overexpression plasmid was produced by GenePharma (Shanghai, China). Cell transfection was performed in rMSCs with Lipofectamine 2000 (Invitrogen, Carlsbad, CA, USA) according to the manufacturer’s protocol. The rMSCs were divided into the following groups: (1) si-SHP/hypoxia group (rMSCs were transfected with si-SHP, followed by hypoxia intervention for 24 and 48 h); (2) miR-127-5p inhibitor/induction group (rMSCs were transfected with the miR-127-5p inhibitor, followed by induction of chondrogenic differentiation for 7 and 14 days); (3) miR-127-5p + hypoxia/induction group (rMSCs were transfected with miR-127-5p mimics, followed by hypoxia intervention and induction of chondrogenic differentiation for 14 days); (4) DNM3OS/induction group (rMSCs were transfected with the DNM3OS overexpression plasmid, followed by induction of chondrogenic differentiation for 14 days); (5) si-DNM3OS + hypoxia/induction group (rMSCs were transfected with si-SHP, followed by hypoxia intervention and induction of chondrogenic differentiation for 14 days).

### Western blot

Total protein was extracted with radio-immunoprecipitation assay (RIPA) lysis buffer (Boster Biological Technology, Inc., Wuhan, China), and the protein concentration in each extract determined by a BCA assay (Beyotime, China). An equal amount of protein from each extract was separated by SDS-PAGE electrophoresis, and the protein bands were transferred onto PVDF membranes (Millipore, Billerica, MA, USA). Next, the membranes were incubated with 10% non-fat dried milk in TBST overnight at 4 °C and subsequently incubated with primary antibodies against COL1A1 (ab270993; 1:1000 diluted), COL2A1 (ab188570; 1:2000 diluted), SOX9 (ab185966; 1:2000 diluted), ACAN (ab36861; 1:1000 diluted), HIF-1α (ab179483; 1:1000 diluted), SHP (ab32559; 1:1000 diluted), ERRγ (ab128930; 1:1000 diluted), BMP2 (ab214821; 1:1000 diluted), p-SMAD1 (ab226821; 1:1000 diluted), p-SMAD2 (ab280888; 1:1000 diluted), GREM2 (ab228736; 1:1000 diluted), and β-actin (ab8226; 1:1000 diluted) overnight at 4 °C. After being washed 3 times with TBST (5 min per wash), the membranes were incubated with a HRP-conjugated secondary antibody for 2 h at room temperature. Subsequently, the protein bands were detected by enhanced chemiluminescence and band staining intensities were quantified using Image software.

### Immunofluorescence

For immunofluorescence staining of GREM2, rMSCs from the different groups were washed with PBS (3 times). Then, they were fixed with paraformaldehyde (4%) for 15 min, and permeabilized with Triton X-100 (0.1%) for 5 min at room temperature. After finishing blocking in 0.1% BSA, the rMSCs were incubated with the primary antibody against GREM2 overnight at 4 °C, followed by incubation with CY3-conjugated goat anti-rabbit IgG (1:100 dilution; Boster Biological Technology) for 1 h at room temperature. Then it was treated with DAPI for nuclear staining. After being rinsed again with PBS, the rMSCs were imaged using a fluorescence microscope at 200 × magnification.

### Reverse transcription quantitative PCR (RT-qPCR)

RNA was extracted from cells using TRIzol reagent (Invitrogen). Reverse transcription was performed according to instructions of M-MLV Reverse Transcriptase (Invitrogen). For detection of miR-127-5p, RNA was first transferred into cDNA with a specific reverse transcription primer (5ʹ-CTCA ACT GGT GTC GTG GAG TCG GCA ATT CAG TTG AGA ATC AGA G-3ʹ). Power SYBR Green PCR Master Mix (Applied Biosystems, Foster, CA, USA) was used to perform quantitative PCR on a LightCycler 480 Real-Time PCR system (Roche, Shanghai, China). The PCR conditions consisted of 95 °C for 2 min, followed by 40 cycles of 95 °C for 15 s and 60 °C for 30 s. Table 1 shows the primer sequences used above. Relative levels of gene expression were calculated by the 2^−ΔΔCt^ method with U6 or β-actin serving as an internal control.

**Table 1:** Primer sequences used for RT-qPCR

Gene	Forward (5ʹ—3ʹ)	Reverse (5ʹ—3ʹ)
DNM3OS	CATCCCAGGACTGAAGTCATTT	AATAAGTTGTGGGTCGGTGC
MiR-127-5p	ACACTCCAGCTGGGCTGAAGCTCAGAGGGCTC	CTCAACTGGTGTCGTGGA
SHP	CCCTGTCATCGGAGATGTTG	TGCAAACCGAGGAAATCCAA
GREM2	TGGCACATACAGAGGAGTCA	AGGGACAGTTCACCTACGTT
U6	CTCGCTTCGGCAGCACA	AACGCTTCACGAATTTGCGT
β-actin	CCCATCTATGAGGGTTACGC	TTTAATGTCACGCACGATTTC

### Alcian blue staining assay

For Alcian blue staining, rMSCs from different groups were gently rinsed twice with PBS, and then fixed with methanol for 30 min at − 20 °C. Afterwards, the rMSCs were washed twice again with PBS and then steeped in Alcian blue in 0.1 N HCl for 30 min. Finally, the rMSCs were washed 3 times with distilled water and the stained cells were observed under a microscope at 400 × magnification.

### Luciferase reporter assay

The targets of DNM3OS were predicted by the LncBase Predicted v2 database, which identified miR-127-5p as containing a binding site for DNM3OS. TargetScan (http://www.targetscan.org/vert_72/) predicted *GREM2* as a potential regulatory target of miR-127-5p. For luciferase reporter assays, the wild-type (WT) or mutant 3′-untranslated regions (UTRs) of GREM2 were subcloned into a psiCHECK TM-2 vector (Promega, Madison, WI, USA). Additionally, WT or MUT GREM2 vectors were con-transfected with miR-127-5p or the NC into rMSCs. The GREAM2 3′UTR vector was transfected into rMSCs from the control, induction, and DNM3OS/induction groups. After 48 h of transfection, Firefly and Renilla luciferase activities were quantified. Relative luciferase activity was determined using Renilla luciferase activity as an internal control.

### Statistical analysis

All quantitative data were analyzed using IBM SPSS Statistics for Windows, Version 19.0 (IBM Corp., Armonk, NY, USA). Differences between multiple groups were analyzed by one-way ANOVA followed by Tukey’s post-hoc test. All data are shown as mean ± standard deviation obtained from three independent experiments. A *p*-value < 0.05 was considered to be statistically significant.

## Results

### Expression and transcriptional regulation of miR-127-5p during chondrogenic differentiation of rMSCs under hypoxic conditions

**Fig. 1:**
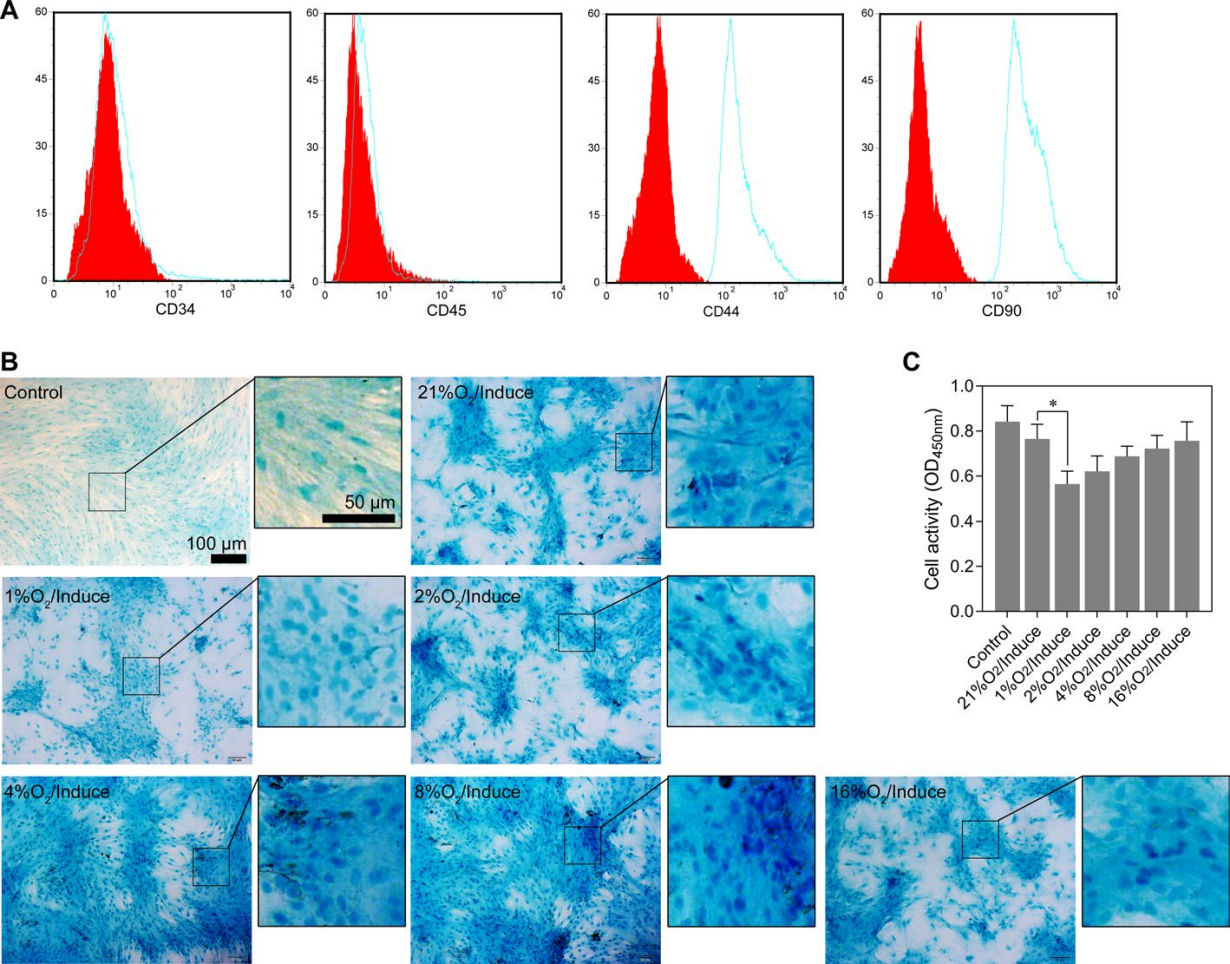
Hypoxia influenced the chondrogenic differentiation of rMSCs. (A) Isolated rMSCs were identified by flow cytometry. **B** The rMSCs were cultured with MCDM under hypoxic conditions (1, 2, 4, 4 8, 16, 21% O_2_) for 14 days. Proteoglycan deposition was shown by Alcian blue staining assays. **C** Cell activity was showed by Cell Counting Kit-8. *p < 0.05

**Fig. 2:**
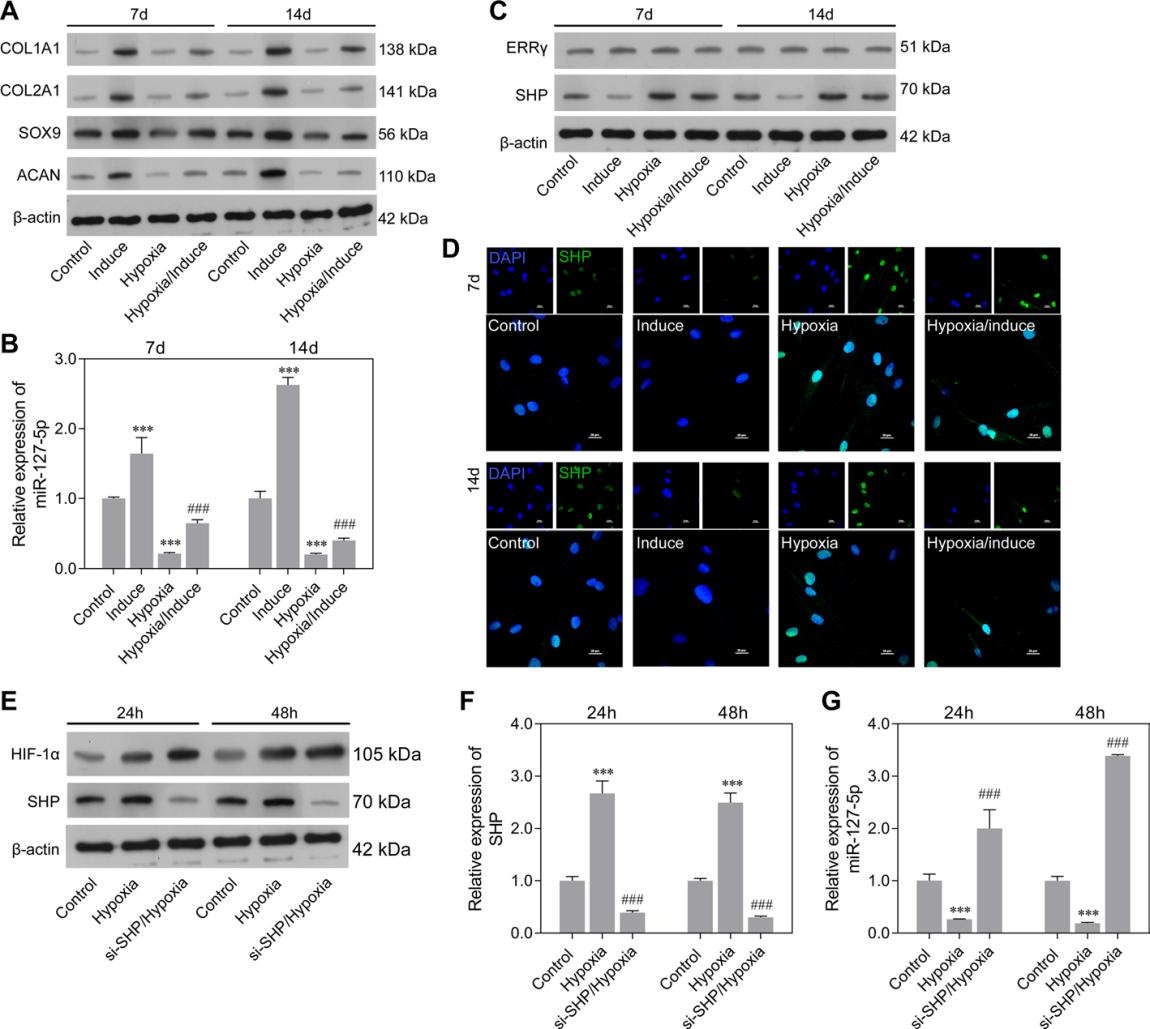
Expression and transcriptional regulation of miR-127-5p during chondrogenic differentiation of rMSCs under hypoxic conditions. The rMSCs were induced to undergo chondrogenic differentiation under hypoxic conditions (1% O_2_) for 7 and 14 days. **A** Expression of COL1A1, COL2A1, SOX9, and ACAN proteins in rMSCs in the control, induction, hypoxia, and hypoxia/induction groups was detected by western blotting. **B** MiR-127-5p expression was determined by quantitative real-time PCR analysis. **C** Transcriptional factors (ERRγ and SHP) were analyzed by western blotting. **D** SHP protein expression was detected by immunofluorescence. rMSCs were transfected with si-SHP, followed by hypoxia intervention for 24 and 48 h. **E** Western blotting was performed to examine the relative levels of HIF-1α and SHP protein expression in rMSCs from the control, hypoxia, and si-SHP/hypoxia groups. Quantitative real-time PCR was used to examine SHP (**F**) and miR-127-5p expression (**G**). Results represent the mean value ± standard deviation of data obtained from three independent experiments. ***p < 0.001, compared with control; ^###^*p* < 0.001, compared with hypoxia

Isolated rMSCs were identified by flow cytometry. The results showed that the cells expressed CD90 and CD44 and did not express hematopoietic markers CD34 and CD45 (Fig. 1A). Chondrogenic differentiation in rMSCs was induced by incubating the rMSCs with MCDM for 14 days, followed by normoxia (21% O_2_) or hypoxia (< 21% O_2_) stimulation. Results of Alcian blue staining showed that 1% O_2_ conditions inhibited chondrogenic differentiation (Fig. 1B) as well as the cell activity (Fig. 1C). Furthermore, we analyzed the levels of chondrogenic differentiation protein markers (COL1A1, COL2A1, SOX9, and ACAN) in normoxic or hypoxic conditions (1% O_2_). As shown in Fig. 2A, our data showed that the protein levels of COL1A1, COL2A1, SOX9, and ACAN were obviously higher in the induction groups than the non-induction groups under either normoxic or hypoxic conditions at 7 and 14 days, suggesting effective induction in rMSCs. Moreover, hypoxic conditions attenuated the chondrogenic differentiation of rMSCs compared with normoxic conditions. Next, we detected the levels of miR-127-5p at 7 and 14 days in rMSCs by RT-qPCR. The results showed that the expression of miR-127-5p was significantly higher in the induction group, but lower in the hypoxia group, compared with the control group at 7 and 14 days. Moreover, the levels of miR-127-5p in the hypoxia/induction group were markedly lower than those in the induction group (Fig. 2B). These data suggested that miR-127-5p might play an important role in the mechanism by which hypoxia suppresses the differentiation of rMSCs. Next, we selected two transcription factors (estrogen related receptor gamma [ERRγ] and small heterodimer partner [SHP]) to analyze the transcriptional regulation of miR-127-5p. Western blotting and immunofluorescence studies showed that the downregulation of SHP in the induction group was reversed by hypoxic conditions at 7 and 14 days, while there was no obvious difference in ERRγ expression between the two groups (Fig. 2C, D). We also found that si-SHP transfection significantly suppressed SHP protein (Fig. 2E) and mRNA expression (Fig. 2F) but upregulated miR-127-5p expression (Fig. 2G) in rMSCs in hypoxic conditions. These results suggested that hypoxia stimulation reduced miR-127-5p levels by promoting SHP expression.

### Downregulation of miR-127-5p suppressed chondrogenic differentiation of rMSCs via SMAD-dependent BMP signaling

**Fig. 3:**
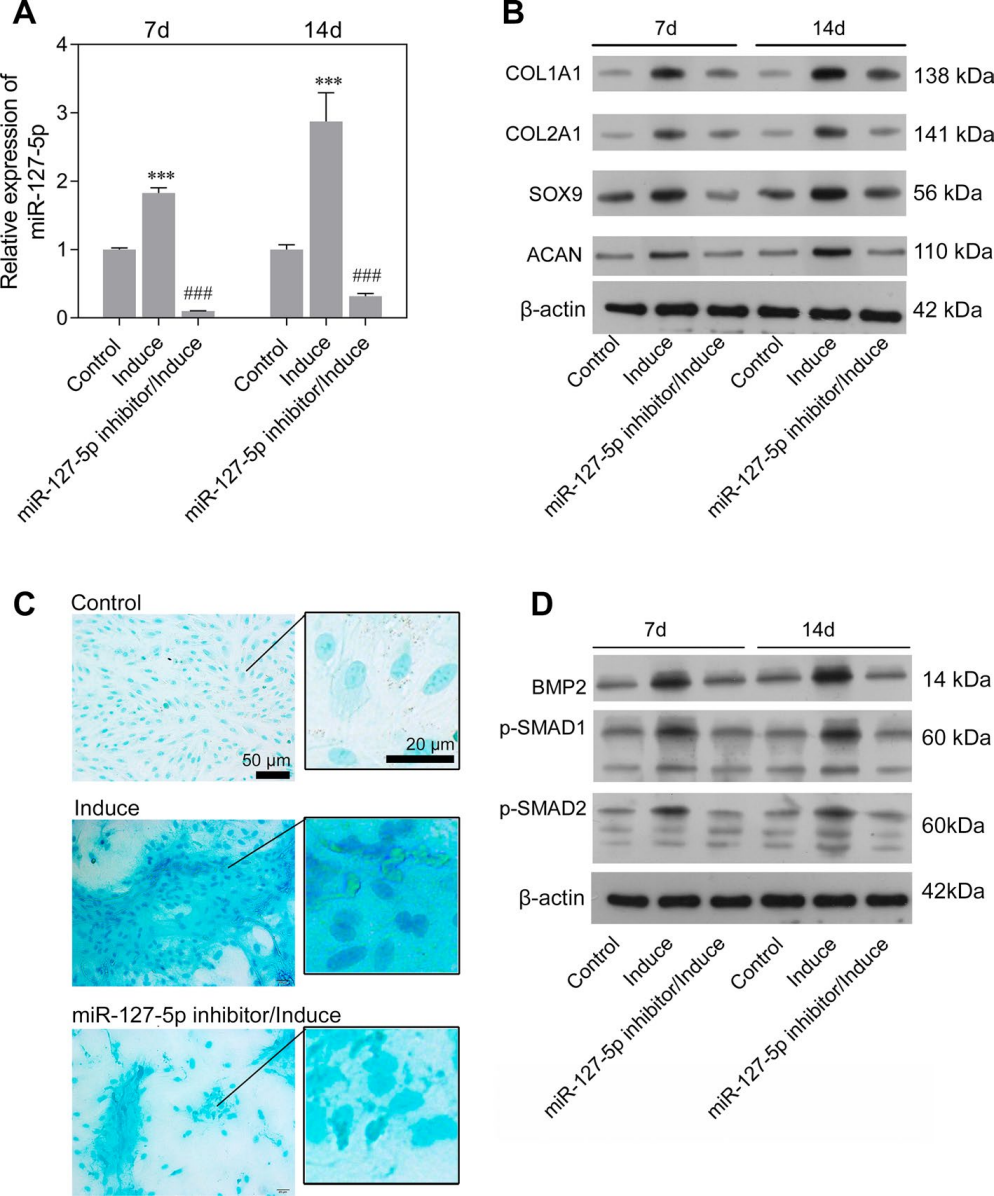
Downregulation of miR-127-5p suppressed chondrogenic differentiation of rMSCs via SMAD-dependent BMP signaling. The rMSCs were transfected with an miR-127-5p inhibitor, followed by induction of chondrogenic differentiation for 7 and 14 days. **A** MiR-127-5p expression was determined by quantitative real-time PCR analysis. Results are expressed as mean ± standard deviation of data obtained from three independent experiments. ***p < 0.001, compared with control; ^###^*p* < 0.001, compared with induction group; **B** Levels of COL1A1, COL2A1, SOX9, and ACAN protein expression were detected in rMSCs. **C** Images from Alcian blue staining assays of rMSCs. **D** Western blotting was performed to measure the levels of BMP2, p-SMAD1, and p-SMAD2 protein expression in rMSCs

To investigate how miR-127-5p functions in the chondrogenic differentiation of rMSCs, an miR-127-5p inhibitor was transfected into rMSCs, followed by induction with MCDM for 7 or 14 days. Control cells consisted of rMSCs transfected with the NC. RT-qPCR analysis revealed that the levels of miR-127-5p in the induction group rMSCs were higher than those in the controls (Fig. 3A). Moreover, transfection with the miR-127-5p inhibitor markedly inhibited the increase in miR-127-5p levels (Fig. 3A). There were also obviously elevated levels of COL1A1, COL2A1, SOX9, and ACAN proteins after induction with MCDM for 7 and 14 days, but all those increases were eliminated by transfection with the miR-127-5p inhibitor (Fig. 3B). Alcian blue staining assays were performed to examine the proteoglycans present during chondrogenic differentiation of rMSCs. As shown in Fig. 3C, induction with MCDM promoted proteoglycan deposition, and that effect was reversed by the miR-127-5p inhibitor. Furthermore, results of western blot analyses indicated that miR-127-5p knockdown suppressed the upregulation of osteo/chondrogenic markers (BMP-2, p-SMAD1/2) in the induction group (Fig. 3D). Taken together, our data suggested that miR-127-5p promotes chondrogenic differentiation of rMSCs via SMAD-dependent BMP signaling.

### GREM2 was a target of miR-127-5p

**Fig. 4:**
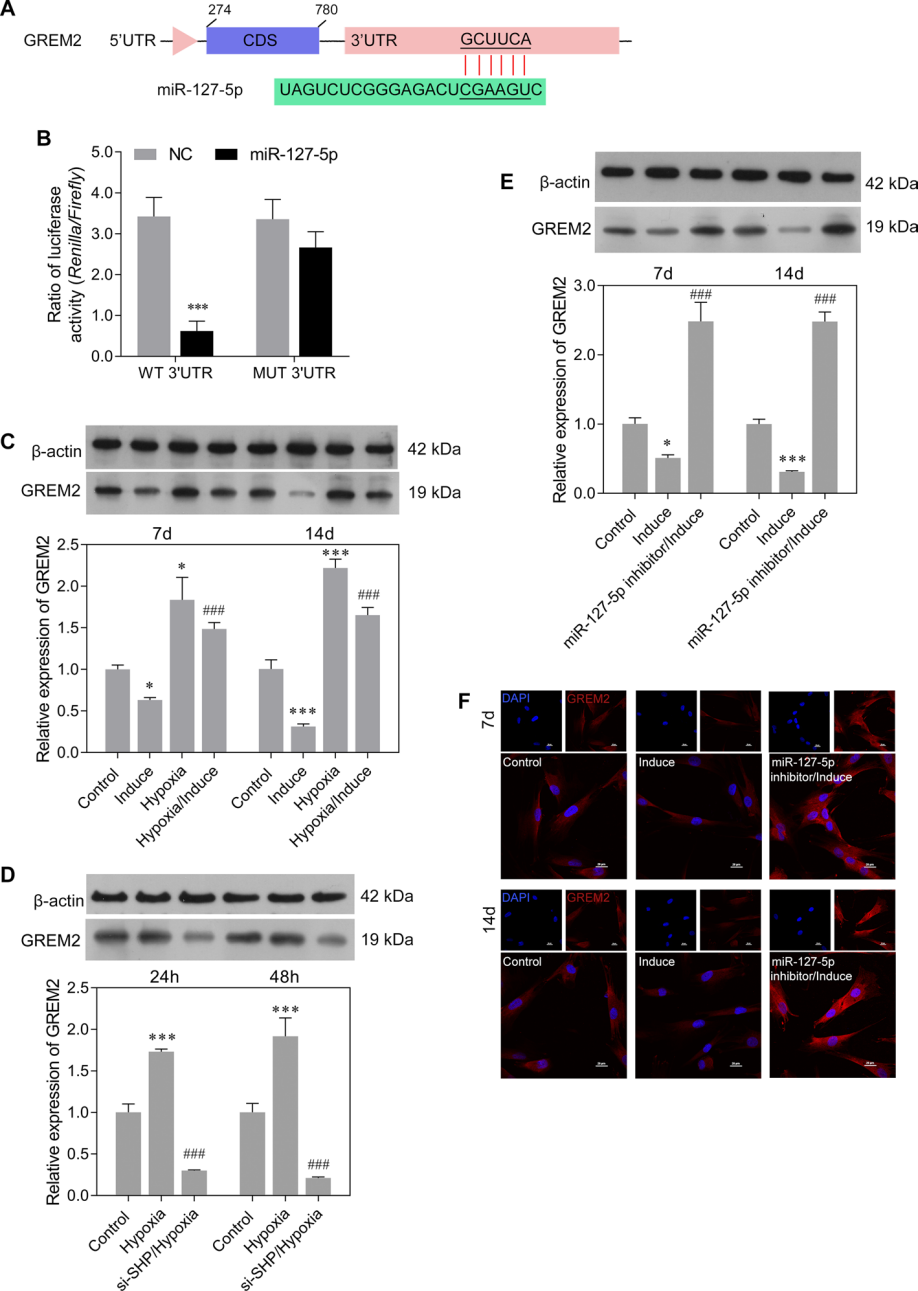
GREM2 was a target of miR-127-5p. **A** Nucleotide sequences of the predicted target site for miR-127-5p in the GREM2 3ʹ-UTR. **B** Luciferase reporter assays were performed to identify direct interaction between miR-127-5p and the GREM2 3ʹ-UTR. Wild type and mutant miR-127-5p target binding sequences in the GREM2 3ʹ-UTR were cloned into a reporter luciferase vector and co-transfected with the synthetized miR-127-5p (or NC) into rMSCs. ***p < 0.001, compared with NC; **C** GREM2 protein expression was measured in rMSCs from the control, induction, hypoxia, and hypoxia/induction groups. **D** GREM2 protein expression was measured in rMSCs from the control, induction, and si-SHP/hypoxia groups. **E** GREM2 protein expression was measured in rMSCs from the control, induction, and miR-127-5p inhibitor/induction groups. **F** GREM2 protein expression in rMSCs was measured by immunofluorescence. *p < 0.05, ***p < 0.001, compared with control; ^#^*p* < 0.05, ^###^*p* < 0.001, compared with hypoxia or induction group

To explore the function of miR-127-5p in chondrogenic differentiation, the potential targets of miR-127-5p were predicted using bioinformatics software as described above. Those results identified a potential binding site for miR-127-5p in the 3ʹ-UTR sequence of GREM2 (Fig. 4A). To test whether miR-127-5p could directly target GREM2 expression, GREM2 3ʹ-UTR fragments were cloned and recombined to a psi-CHECK2 plasmid. Dual luciferase assays showed that miR-127-5p significantly reduced the fluorescence ratio in the WT 3ʹ-UTR and miR-127-5p mimics co-transfection group (Fig. 4B). We next determined the level of GREM2 expression in rMSCs undergoing chondrogenic differentiation under hypoxic conditions. As shown in Fig. 4C, GREM2 expression was obviously downregulated by induction with MCDM for 7 or 14 days in both the normoxia and hypoxia group. Hypoxia treatment notably increased GREM2 expression in the induction group. We also investigated the roles played by SHP and miR-127-5p in regulating GREM2 expression. Those results showed that SHP knockdown suppressed the expression of GREM2 protein under hypoxic conditions (Fig. 4D). In addition, downregulation of miR-127-5p increased GREM2 expression in rMSCs that had been induced with MCDM for 7 or 14 days (Fig. 4E, F). Our results suggested that GREM2 was a direct target of miR-127-5p, and was suppressed during the chondrogenic differentiation of rMSCs.

### Overexpression of miR-127-5p promoted chondrogenic differentiation of rMSCs by regulating GREM2-mediated SMAD-dependent BMP signaling

**Fig. 5:**
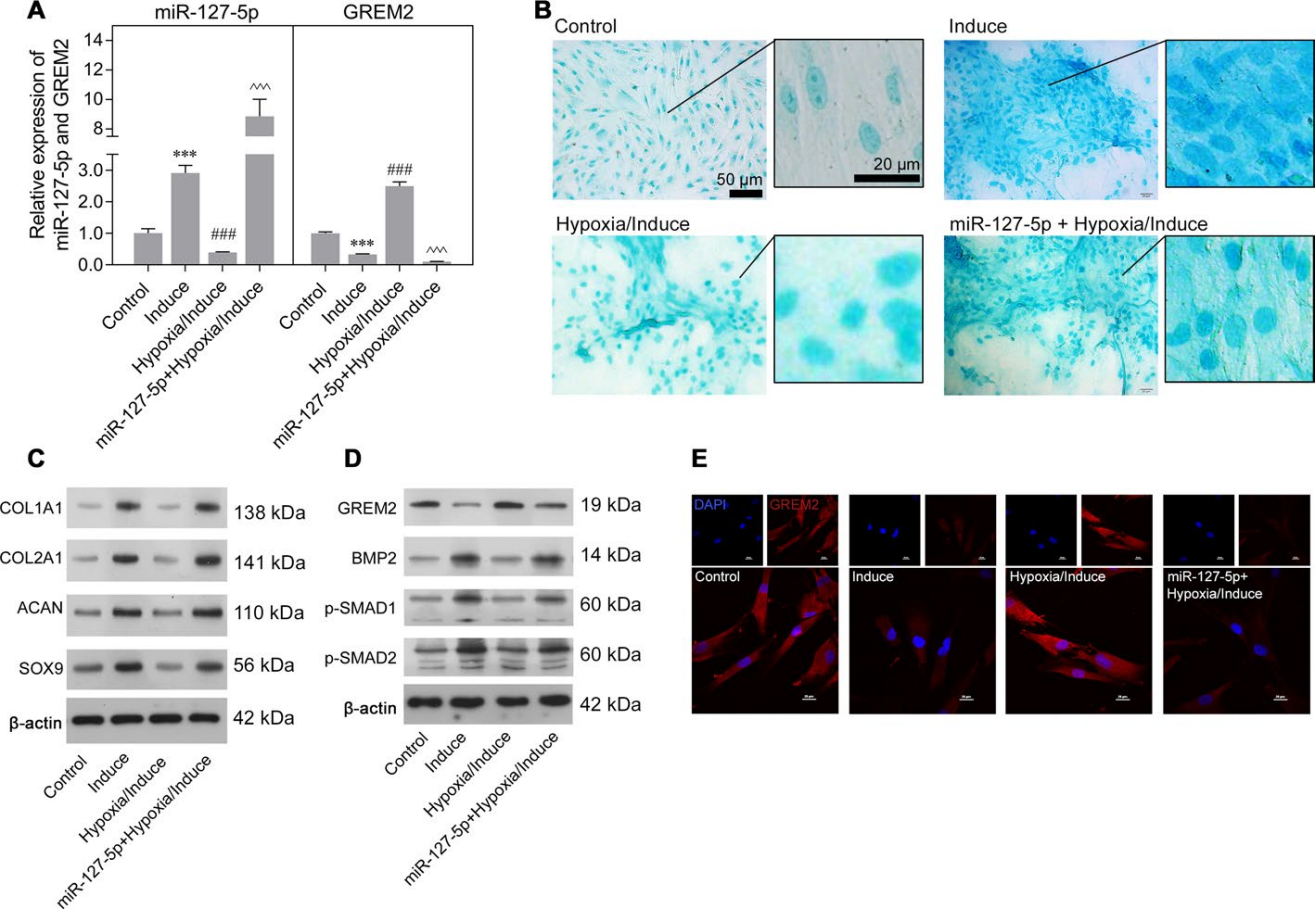
Overexpression of miR-127-5p promoted chondrogenic differentiation of rMSCs by regulating GREM2-mediated SMAD-dependent BMP signaling. The rMSCs were transfected with miR-127-5p mimics, followed by hypoxia intervention (1% O_2_) and induction of chondrogenic differentiation for 14 days. **A** MiR-127-5p and GREM2 expression levels were determined by quantitative real-time PCR analysis. Results are expressed as mean ± standard deviation of data obtained from three independent experiments. ***p < 0.001, compared with control; ^###^*p* < 0.001, compared with induction group; *p* < 0.001, compared with hypoxia/induction; **B** Images from Alcian blue staining assays of rMSCs. **C** Levels of COL1A1, COL2A1, SOX9, and ACAN protein expression were detected in rMSCs. **D** Levels of GREM2, BMP2, p-SMAD1, and p-SMAD2 protein expression in rMSCs were detected by western blotting. **E** GREM2 protein expression in rMSCs was detected by immunofluorescence

Because miR-127-5p was downregulated in rMSCs in the hypoxia/induction group compared with rMSCs in the induction group, we performed gain-of-function assays with rMSCs to investigate the effects of miR-127-5p on the chondrogenic differentiation of rMSCs under hypoxic conditions. Quantitative real-time PCR confirmed the upregulation of miR-127-5p and downregulation of GREM2 in rMSCs from the hypoxia/induction group following transfection with miR-127-5p mimics (Fig. 5A). Alcian blue staining assays indicated that miR-127-5p overexpression reversed the decrease in proteoglycan deposition induced by hypoxic conditions (Fig. 5B). Furthermore, the decreases in expression of chondrogenic differentiation markers (COL1A1, COL2A1, SOX9, and ACAN) in rMSCs from the induction group cultured under hypoxic conditions were reversed by miR-127-5p overexpression (Fig. 5C). We also found that GREM2 expression was upregulated while the levels of osteo/chondrogenic markers (BMP-2, p-SMAD1/2) were downregulated in rMSCs from the induction group cultured under hypoxic conditions, and all those changes could be reversed by miR-127-5p overexpression (Fig. 5D). Immunofluorescence studies further confirmed that the upregulated expression of GREM2 in rMSCs from the induction group cultured under hypoxic conditions could be reversed by miR-127-5p overexpression. (Fig. 5E).

### DNM3OS inhibited chondrogenic differentiation of rMSCs via miR-127-5p regulation of GREM2

**Fig. 6:**
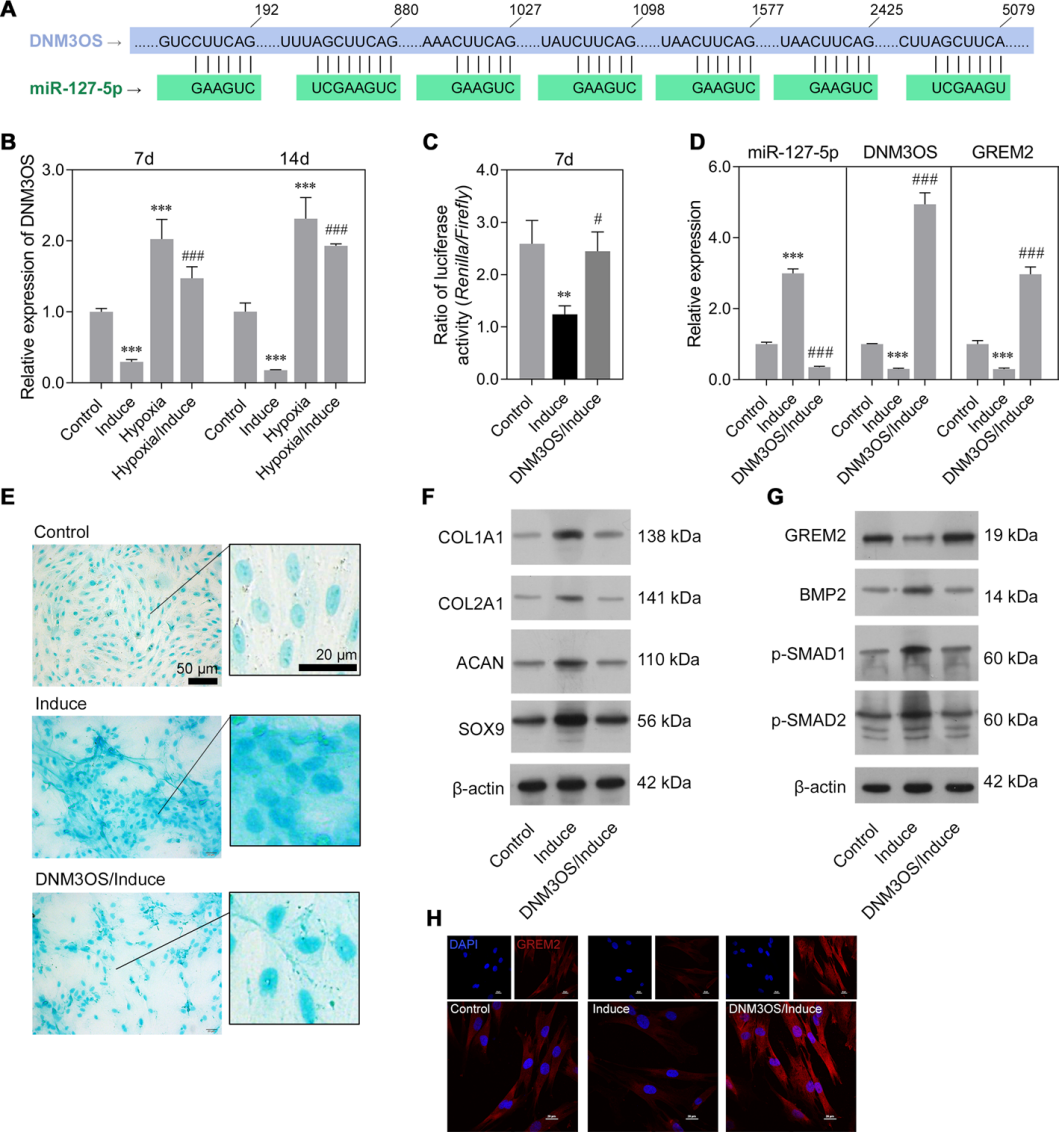
DNM3OS inhibited chondrogenic differentiation of rMSCs via miR-127-5p regulation of GREM2. The rMSCs were transfected with a DNM3OS overexpression plasmid, followed by induction of chondrogenic differentiation for 14 days. Accordingly, the rMSCs were classified into control, induction, hypoxia, hypoxia/induction, and DNM3OS/induction groups. **A** The putative binding site for miR-127-5p in the DNM3OS 3′-UTR is shown. **B** DNM3OS expression was determined by quantitative real-time PCR analysis. **C** A luciferase reporter assay was performed to identify direct action between DNM3OS and the GREM2 3ʹ-UTR. **D** Levels of DNM3OS, miR-127-5p, and GREM2 expression were determined by quantitative real-time PCR analysis. Results are expressed as mean ± standard deviation of data obtained from three independent experiments. **p < 0.01, ***p < 0.001, compared with control; ^#^*p* < 0.05, ^###^*p* < 0.001, compared with induction group; **E** Images from Alcian blue staining assays of rMSCs. (F) Levels of COL1A1, COL2A1, SOX9, and ACAN protein expression were detected in rMSCs. **G** Levels of GREM2, BMP2, p-SMAD1, and p-SMAD2 in rMSCs were detected by western blotting. **H** GREM2 protein expression in rMSCs was measured by immunofluorescence

**Fig. 7:**
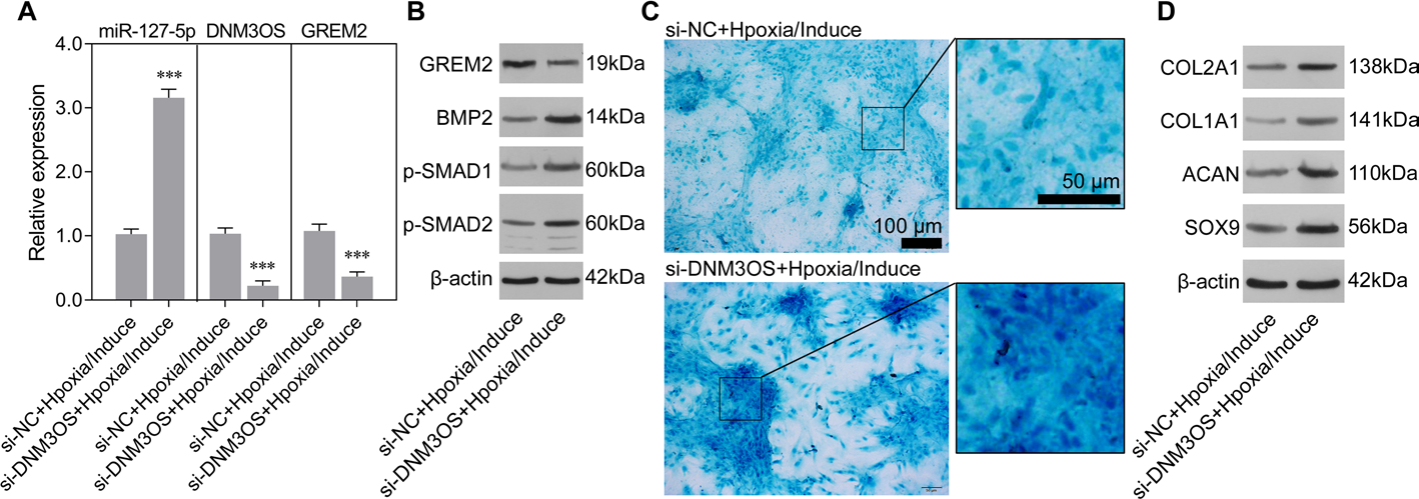
DNM3OS knockdown weakened the inhibitory effect of hypoxia on chondrogenic induction. The rMSCs were transfected with si-DNM3OS, followed by induction of chondrogenic differentiation under hypoxic conditions (1% O_2_) for 14 days. **A** Levels of miR-127-5p, DNM3OS, and GREM2 expression were determined by quantitative real-time PCR analysis. ***p < 0.001, compared with si-NC + hypoxia/induction group. **B** Levels of GREM2, BMP2, p-SMAD1, and p-SMAD2 in rMSCs were detected by western blotting. **C** Images from Alcian blue staining assays of rMSCs. (D) Levels of COL1A1, COL2A1, SOX9, and ACAN protein expression were detected in rMSCs. si-NC: negative control

An analysis performed on the LncBase Predicted v2 database predicted miR-127-5p to be the interactive object of DNM3OS (Fig. 6A). Similar to GREM2 expression, DNM3OS expression was downregulated in the induction group and upregulated in the hypoxia group, compared to the control group. Moreover, stimulation with hypoxic conditions reversed the decrease in DNM3OS expression in the induction group (Fig. 6B). Next, a luciferase reporter assay was performed to verify whether DNM3OS regulated GREM2 by acting as an endogenous sponge of miR-127-5p. As illustrated in Fig. 6C, the luciferase activity of the GREM2 wild-type reporter was strongly suppressed in the induction group, and that reduction was reversed by DNM3OS overexpression. Quantitative real-time PCR further confirmed that the downregulation of DNM3OS and GREM2 and upregulation of miR-127-5p in the induction group rMSCs could be reversed by DNM3OS overexpression (Fig. 6D). Alcian blue staining assays showed that DNM3OS overexpression suppressed the proteoglycan deposition in rMSCs caused by induction with MCDM (Fig. 6E). Western blot studies showed that the upregulation of chondrogenic differentiation markers (COL1A1, COL2A1, SOX9, and ACAN) (Fig. 6F) as well as the decrease in GREM2 expression and upregulation of osteo/chondrogenic markers (BMP-2, p-SMAD1/2) (Fig. 6G) caused by induction with MCDM could all be reversed by DNM3OS overexpression. Immunofluorescence studies also confirmed that the decrease in GREM2 expression in rMSCs from the induction group could be reversed by DNM3OS overexpression. (Fig. 6H). Conversely, DNM3OS knockdown significantly increased the expression of miR-127-5p, while it reduced that of GREM2 (Fig. 7A). Western blot studies showed that the protein levels of BMP-2 and p-SMAD1/2 were increased by DNM3OS knockdown. Moreover, Alcian blue staining assays showed that DNM3OS knockdown attenuated the inhibitory effect of hypoxia on proteoglycan deposition in rMSCs (Fig. 7B). The protein levels of COL1A1, COL2A1, SOX9, and ACAN were also increased in the DNM3OS knockdown group (Fig. 7C). The above results suggested that DNM3OS plays an important role in hypoxic chondrogenic differentiation.

## Discussion

Degradation of articular cartilage is the characteristic pathological feature of OA; however, such cartilage damage can be repaired by chondrogenesis, mainly resulting from MSC condensation and differentiation into chondrocytes [31]. In the present study, we found that miR-127-5p expression was significantly increased during chondrogenic induction of rMSCs under normoxic conditions, and those increases were notably attenuated in rMSCs induced under hypoxic conditions. A series of recent studies has indicated that hypoxia promotes osteogenic differentiation and chondrogenic differentiation of MSCs [32–34]. However, in the present study we found that hypoxia inhibits chondrogenic differentiation. Different hypoxia conditions may be the main reason for this result. In previous studies the cells were cultured under hypoxia (2% O_2_) [35], while we performed hypoxia with 1% O_2_. The two concentrations of O_2_ both induced expression of HIF-1α, but the resultant damage may be different. A previous study emphasized that O_2_ is a critical parameter for chondrogenic differentiation [36]. Subsequently, we found that the rMSCs’ survival was significantly inhibited by hypoxia (1% O_2_), which may seriously affect cell differentiation. Certainly, there are other factors influencing the chondrogenic differentiation of rMSCs, such as the frequency of medium change and induction time, and these factors need more research to investigate whether they affect the role of hypoxia in chondrogenic differentiation. Further in vitro experiments revealed that downregulation of miR-127-5p reduced the production of proteoglycans and suppressed the chondrogenic differentiation of rMSCs, as reflected by decreased levels of chondrogenic differentiation markers (COL1A1, COL2A1, SOX9, and ACAN) [37]. Furthermore, a study reported that BMP2 plays a vital role in inducing the osteogenic differentiation of MSCs [38]. Dong et al. [39] reported that the BMP2/SMAD signaling pathway is crucial for osteogenic differentiation, and that silicon stimulates collagen type 1 and osteocalcin synthesis via the BMP2/SMAD/RUNX2 pathway. Furthermore, Li et al. [40] reported that the BMP2/SMAD/AKT/RUNX2 signaling pathway is the key regulator of osteoblastogenesis. Here, we found that miR-127-5p knockdown reduced the levels of BMP2, p-SMAD1, and p-SMAD2 in rMSCs induced to undergo chondrogenic differentiation. When miR-127-5p was overexpressed in rMSCs induced to undergo chondrogenic differentiation under hypoxic conditions, the opposite results were obtained with miR-127-5p knockdown. These data indicated that miR-127-5p plays positive roles in the chondrogenic differentiation of rMSCs under both normoxic and hypoxic conditions. Consistent with our findings, miR-127-5p is known to act in conjunction with its target (PTEN) to promote osteogenesis via the PTEN/AKT signaling pathway [41]. It was also reported that miR-127-5p promotes cartilage differentiation and reduces the hypertrophy of BMSCs found in cartilage [25].

Generally, miRNAs have important functions in stem cell self-renewal and differentiation by either inducing degradation of their target gene mRNA molecules or inhibiting mRNA translation via binding to complementary target sequences [42–44]. Previous studies showed that miR-127-5p targets several osteogenesis-related genes, including *MMP-13* [22, 45] and osteopontin (*OPN*) [24]. In this study, luciferase reporter assays and western blot analyses consistently demonstrated that miR-127-5p suppressed GREM2 expression in rMSCs undergoing chondrogenic differentiation. Moreover, GREM2 expression was upregulated after hypoxia stimulation but downregulated during chondrogenic differentiation of rMSCs. In line with our data, recombinant human GREM2 was previously shown to suppress phosphorylation of SMAD1/5 induced by BMP2 and the osteogenic differentiation of human tenocytes in vitro [46]. Knockdown of GREM2 is known to increase the BMP-2-induced osteogenic differentiation of human bone marrow-derived MSCs via the BMP-2/SMAD/RUNX2 pathway [27]. This evidence led us to speculate that GREM2 might be the downstream regulator involved in the mechanism by which miR-127-5p promotes chondrogenic differentiation of rMSCs under hypoxic conditions.

Furthermore, lncRNA DNM3OS has been shown to act as an endogenous sponge of miR-127-5p to upregulate GREM2 expression during induced chondrogenic differentiation under hypoxic conditions. Our functional experiments indicated that DNM3OS overexpression could suppress proteoglycan deposition, upregulate GREM2 expression, and reduce the expression of chondrogenic differentiation markers (COL1A1, COL2A1, SOX9, and ACAN) and osteo/chondrogenic markers (BMP-2, p-SMAD1/2) during induced chondrogenic differentiation. These data suggested that DNM3OS plays a negative role in chondrogenic differentiation of rMSCs. Similarly, a study revealed that the *DNM3OS* promoter containing regulatory elements exerts effects on chondrogenesis [47]. Ai et al. [48] reported that DNM3OS expression was downregulated in OA patients and DNM3OS promotes proliferation of chondrocytes. As the regulator of how hypoxia affects chondrogenic differentiation, HIF-1α was shown to potentiate BMP2-induced cartilage formation and inhibit endochondral ossification during ectopic bone/cartilage formation [34]. Persistent exposure to hypoxia was shown to downregulate CBFA-1/Runx2, osteocalcin, and type I collagen expression in MSCs [16]. On the other hand, hypoxic conditions were shown to increase chondrogenesis in synovium-derived MSCs [49]. Here, our data indicated that hypoxic stimulation suppressed chondrogenic differentiation.

## Conclusions

In conclusion, our study revealed that miR-127-5p might serve as an important positive regulator during chondrogenic differentiation of rMSCs by targeting GREM2 under hypoxic conditions. A further analysis showed that DNM3OS functions as a negative regulator of chondrogenic differentiation by positively regulating GREM2 expression via sponging miR-127-5p. The DNM3OS/miR-127-5p/GREM2 pathway might represent a novel therapeutic target for treating cartilage degenerative disorders, including OA.

## Data Availability

Can be provided upon reasonable request.

## Abbreviations

rMSCs: Rat mesenchymal stem cells
OA: Osteoarthritis
GREM2: Gremlin2
BMP: Bone morphogenic protein
BMSCs: Bone marrow mesenchymal stem cells
ceRNA: Competing endogenous RNA
DNM3OS: Dynamin 3 opposite strand
TGF-β: Transforming growth factor-β
FBS: Fetal bovine serum
MCDM: Mesenchymal stem cell chondrogenic differentiation medium
SHP: Small heterodimer partner
si-SHP: Small interfering RNA targeting SHP
NC: Negative control
BSA: Bovine serum albumin
DAPI: 4ʹ,6-Diamidino-2-phenylindole
ERRγ: Estrogen related receptor gamma
SMAD: Drosophila mothers against decapentaplegic protein
OPN: Osteopontin
HIF-1: Hypoxia inducible factor-1
